# Association of preoperative systemic Immune-inflammation Index and Prognostic Nutritional Index with survival in patients with Upper Tract Urothelial Carcinoma

**DOI:** 10.7150/jca.44915

**Published:** 2020-07-25

**Authors:** Yangqin Zheng, Dongdong Yu, Zhixian Yu, Dewei Zhao, Yuming Chen, Wu Chen, Yeping Li, Binwei Lin, Xiaomin Gao

**Affiliations:** 1 Department of Hematology, The Third Clinical Institute Affiliated to Wenzhou Medical University, People's Hospital of Wenzhou, Wenzhou, Zhejiang province, 325006, P.R. China.; 2 Department of Andrology, The First Affiliated Hospital of Wenzhou Medical University, Wenzhou, Zhejiang province, 325006, P.R. China.; 3 Department of Urology, The First Affiliated Hospital of Wenzhou Medical University, Wenzhou, Zhejiang province,325006, P.R. China.; 4 Department of Urology, Wenzhou Central Hospital, Wenzhou, Zhejiang province, 325200, P.R. China.; 5 Department of Urology, Affiliated Hospital of Yangzhou University, Yangzhou, Jiangsu province, 225001, P.R. China.; 6 Department of Urology, The Third Clinical Institute Affiliated to Wenzhou Medical University, People's Hospital of Wenzhou, Wenzhou, Zhejiang province, 325006, P.R. China.; 7 Department of Urology, Rui'an People's Hospital, The Third Affiliated Hospital of the Wenzhou Medical University, Wenzhou, Zhejiang province, 325200, P.R. China.; 8 Department of Urology, Changhai Hospital, Second Military Medical University, Shanghai, 200433, P.R. China.

**Keywords:** upper tract urothelial carcinoma, systemic immune-inflammation index, prognostic nutritional index, prognosis

## Abstract

**Background:** Both systemic inflammation response and malnutrition are closely related to poor prognosis in patients with certain types of solid tumor. This study investigated the prognostic value of the preoperative combination of systemic immune-inflammation index and prognostic nutritional index (SII-PNI) in patients with upper tract urothelial carcinoma (UTUC) undergoing radical nephroureterectomy (RNU).

**Methods:** The predictive ability of SII-PNI was developed and further validated in a cohort of 525 UTUC patients (253 in the training cohort and 272 in the validation cohort) who received RNU.

**Results:** Survival analysis indicated that a SII ≥672.44 was significantly associated with worse overall survival (OS), cancer-specific survival (CSS), and recurrence-free survival (RFS) while a PNI ≥47.83 was associated with better survival outcomes (All *P*-values < 0.05). The combination of simultaneously SII ≥672.44 and PNI <47.83 was a powerful independent risk factor for OS, CSS, and RFS (*P* < 0.05). The SII-PNI had the largest area under the curve (AUC) compared to that for SII or PNI alone and other clinical factors, indicating its superior for predicting survival. In addition, the incorporation of the SII-PNI into established nomograms or current clinical parameters such as pathologic T stage and N stage, achieved higher c-indexes or larger AUC than without, indicating that adding SII-PNI helped predict prognosis. All results were found in the training cohort and confirmed in the validation cohort.

**Conclusions:** SII-PNI was a strong independent predictor of UTUC patients after RNU.

## Introduction

Despite its status as a relatively uncommon disease, the postoperative survival outcome of upper tract urothelial carcinoma (UTUC) remains unsatisfactory even after radical nephroureterectomy (RNU) with bladder cuff excision. UTUC is remarkably aggressive that a large number of patients will inevitably experience tumor recurrence, metastasis, or even death after surgery 1, 2. The five-year cancer-specific survival (CSS) of UTUC patients ranges from 50-80% following surgery 3, 4. Many preoperative and postoperative factors have been identified as risk predictors of UTUC for patient' risk stratification in order to provide optimal and timely treatment strategies. Traditional risk biomarkers, including pathologic T stage, lymph node status, distant metastasis, tumor size and grade, and patient age, are commonly adopted to assess the prognosis of UTUC in clinical decision-making, although urological outcomes have not significantly improved in the last three decades 5. Therefore, more accurate predictors of survival outcomes after RNU are required.

An increasing number of studies have evaluated the impact of inflammatory and immune variables, including neutrophil-to-lymphocyte ratio (NLR), platelet-to-lymphocyte ratio (PLR), and monocyte-to-lymphocyte ratio (MLR), on the prognosis of UTUC 6-8. However, these inflammation-based biomarkers were too simple, consisting of only two circulating immune cells. Furthermore, the potential role of NLR in patients with UTUC remains controversial 7. Recently, the systemic immune-inflammation index (SII), based on the counts of neutrophils, platelets, and lymphocytes, was developed and had been identified as a powerful and accurate predictor in several solid tumors, including osteosarcoma, esophageal squamous cell carcinoma, lung cancer, and UTUC 6, 9-11. Preoperative malnutritional status is also closely related to postoperative survival outcomes in cancer patients 12. Recently, several studies reported the potential role of novel indicators based on inflammatory and nutritional variables in patients with cancer 13-15. The prognostic nutritional index (PNI), which is calculated from serum albumin level and lymphocyte counts, is one parameters that reflects preoperative nutritional status and has also been validated as an effective prognostic factor in various cancers, including UTUC 12,13. Two studies suggested that the combination of SII and PNI may help identify osteosarcoma and esophageal squamous cell carcinoma patients with poor prognosis 9, 10; however, its independent association with UTUC patients' survival is not reported. This study aimed to explore the associations of the preoperative SII, NLR, PLR, MLR, and PNI with clinicopathologic variables and to identify the predictive ability of individual or combined indicators in patients with UTUC following RNU.

## Materials and Methods

### Patients and study design

Between March 2006 and August 2015, 310 eligible patients with diagnosed UTUC (pathological T1-4N0-1M0) at the First Affiliated Hospital of Wenzhou Medical University were retrospectively enrolled as the training cohort. In addition, 323 patients with UTUC from The Third Clinical Institute Affiliated to Wenzhou Medical University, People's Hospital of Wenzhou diagnosed between July 2004 and December 2016 were retrospectively enrolled as the validation cohort. The exclusion criteria were as follows: (1) patients who underwent palliative surgery instead of RNU; (2) patients with kidney transplantation before surgery; (3) patients with evidence of metastatic disease at the time of surgery; (4) patients with incomplete preoperative medical information; and (5) patients with relevant comorbidities affecting systemic inflammatory response markers (i.e., chronic liver disease, immunosuppression, cytotoxic medications, leukemia, lymphoma, autoimmune diseases, and chronic inflammatory diseases). None of the enrolled patients were treated with neoadjuvant chemotherapy, radiotherapy, or any other anti-tumor therapy. This study was approved by the ethics committee of the First Affiliated Hospital of Wenzhou Medical University and The Third Clinical Institute Affiliated to Wenzhou Medical University, People's Hospital of Wenzhou and was conducted according to the Declaration of Helsinki. All patients agreed to participate in our study and provided written informed consent.

### Definitions of the systemic immune-inflammation index, prognostic nutritional index, and systemic immune cell prognostic score

SII and PNI were defined as follows: SII = (neutrophil×platelet)/lymphocyte; PNI = albumin concentration (g/L) + 5× total lymphocyte count (10^9^/L). NLR, PLR, and MLR were calculated as the cell ratios of neutrophil/lymphocyte, platelet/lymphocyte, and monocyte/lymphocyte, respectively. The optimal cutoff values of SII, PNI, NLR, PLR, and MLR in the training cohort were determined by receiver operating characteristic (ROC) curves analysis with overall survival (OS) as the endpoint.

### Statistical analysis

All data were analyzed using IBM SPSS Statistics for Windows, (version, 25.0, IBM Corp., Armonk, NY) and R software (Version 3.6.0) with the packages rms, Hmisc, and ggplot. Comparisons between variables were evaluated by Student's t-tests (for normally-distributed continuous variables), Mann-Whitney U test (non-normally distributed data), Pearson's chi-square tests, or Fisher's exact tests. The Kaplan-Meier method was used to estimate the differences in survival rates. Univariate and multivariate Cox proportional hazards regressions were conducted to evaluate the prognostic significance of each variable with respect to OS, CSS, and RFS. The significant predictors in multivariate analysis were included in the nomogram construction. The performance and predictive ability of the nomogram were evaluated by calibration plot, concordance index (c-index), and ROC. All *P*-values were two-tailed, and *P* < 0.05 was considered statistically significant.

## Results

### Basic patient characteristics

**Figure 1:**
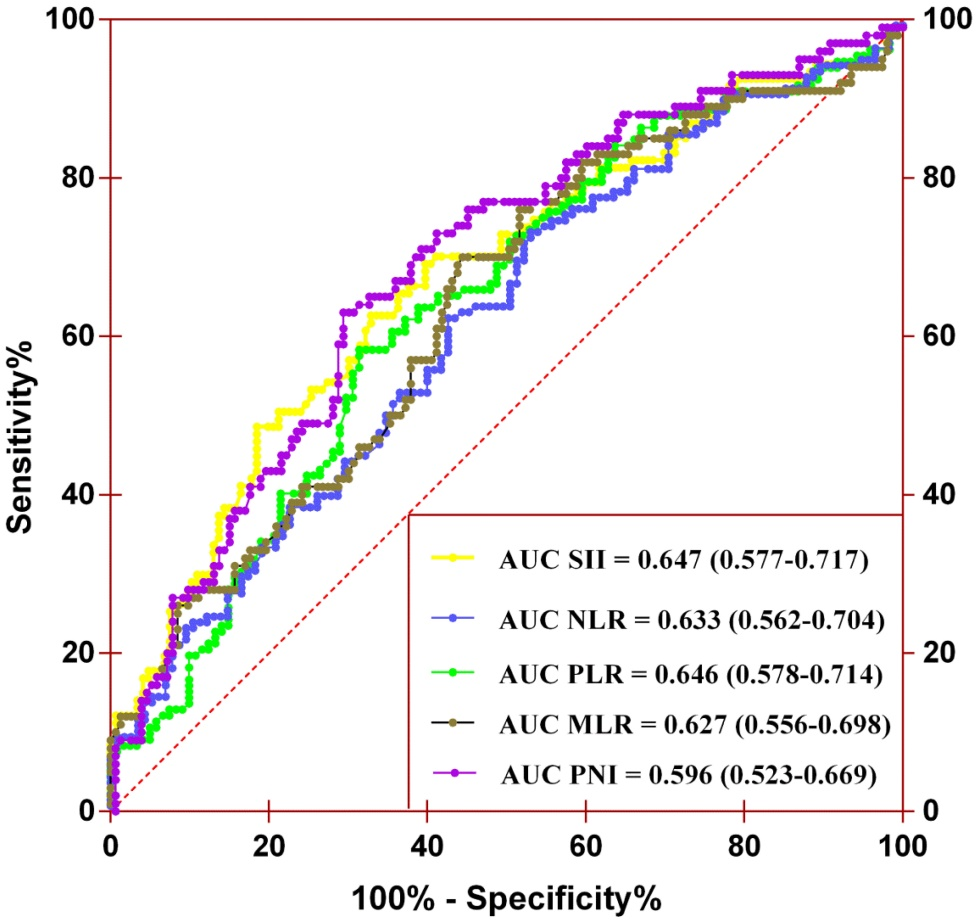
Determination of the optimal cutoff values for SII, NLR, PLR, MLR, and PNI by performing the ROC analysis.

A total of 253 and 272 patients were included in the training and validation cohorts, respectively. The baseline characteristics of the included cases are summarized in Table S1 and Table 1. The training cohort consisted of 180 men (71.1%) and 73 women (28.9%) with a mean age of 67.6 ± 10.5 years. The median follow-up duration was 33.8 (interquartile range [IQR] 16.7-64.4) months. During the follow-up, 93 (36.8%) patients died, 73 (28.9%) of which died of cancer, and 101 (39.9%) patients experienced tumor recurrence after surgery. The validation cohort consisted of 182 men (66.9%) and 90 women (33.1%). The mean age and median follow-up duration were 65.9 ± 10.3 years and 44.6 (IQR 26.8-65.3) months, respectively. During the follow-up, 85 (31.3%) patients died, 66 (24.2%) of whom died of cancer, and 90 (33.1%) patients experienced tumor recurrence after surgery. There were significant differences between the two cohorts in BMI, surgical approach, anemia, CKD stage, and adjuvant therapy (all *P*-values <0.05). The remaining factors were comparable between the two cohorts (Table S1). The optimal cutoff values of SII, PNI, NLR, PLR, and MLR were 672.44, 47.83, 2.53, 126.88 and 0.35 (Figure 1). The area under the curve (AUC) values of SII, PNI, NLR, PLR, and MLR were 0.647 (0.577-0.717), 0.596 (0.523-0.669), 0.633 (0.562-0.704), 0.646 (0.578-0.714), and 0.627 (0.556-0.698), respectively.

Table 1 shows the correlations between inflammatory-based biomarkers (SII and PNI) and patient clinicopathologic parameters in the two cohorts. In the training cohort, SII and PNI were closely related to age, American Society of Anesthesiologists (ASA) grade, body mass index (BMI), surgical approach, NLR, PLR, MLR, anemia, hypoproteinemia, chronic kidney disease (CKD) stage, multifocality, pathologic T stage, N stage, lymphovascular invasion (LVI), and adjuvant therapy (All *P*-values < 0.05). In the validation cohort, SII and PNI were significantly correlated with age, hydronephrosis, surgical approach, NLR, PLR, MLR, anemia, hypoproteinemia, tumor size, pathologic T stage, N stage, tumor grade, and LVI (All *P*-values < 0.05).

### Association with UTUC prognosis

**Figure 2:**
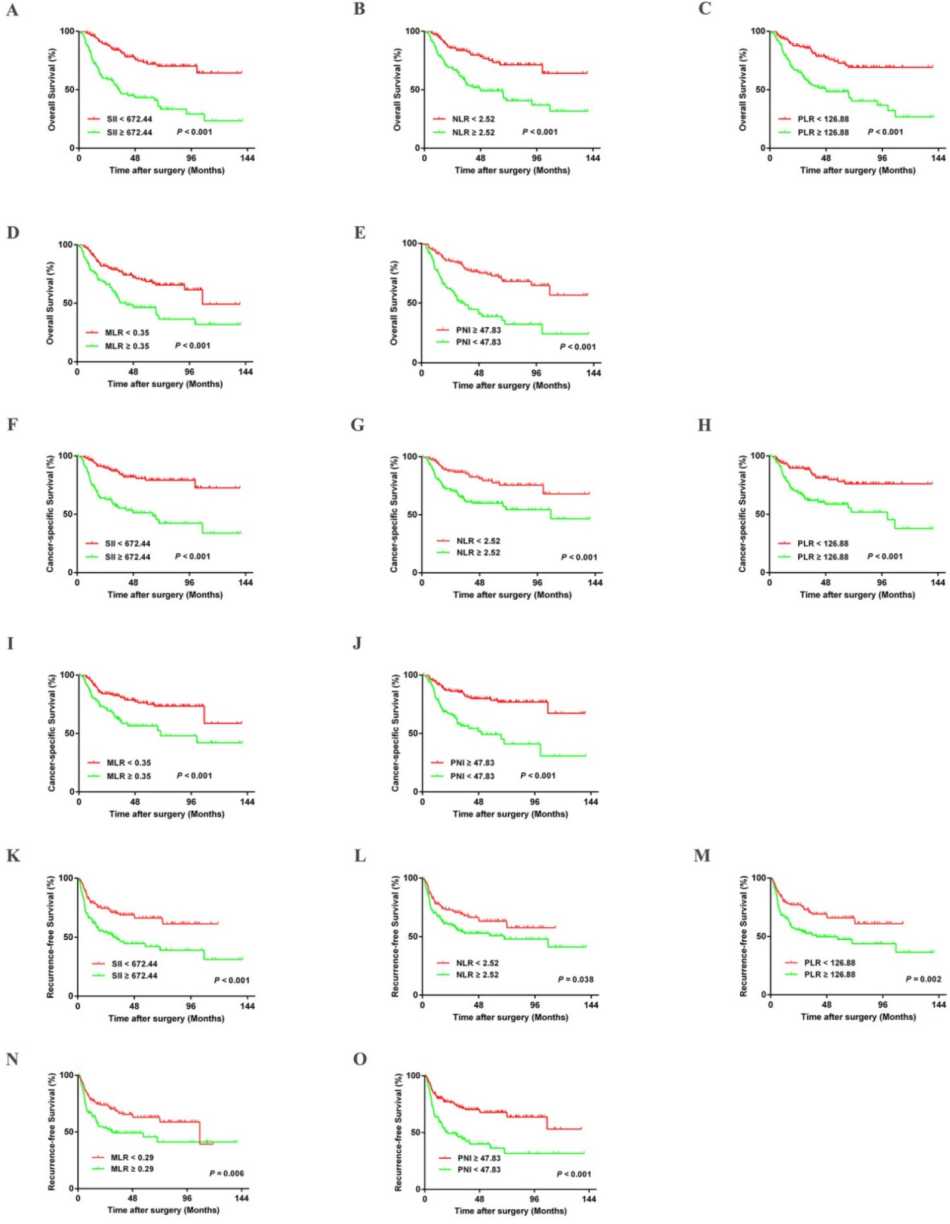
Kaplan-Meier curves for OS, CSS, and RFS in UTUC patients stratified by SII, NLR, PLR, MLR, and PNI in the training cohort.

Kaplan-Meier survival analysis indicated that patients in the training cohort with higher SII, NLR, PLR, and MLR had poorer OS, CSS, and RFS (All *P*-values < 0.05) (Figure 2). Lower OS, CSS, and RFS were observed in patients with lower PNI (All *P*-values < 0.05) (Figure 2). These results were confirmed in the validation cohort (Figure S1). Furthermore, univariate and multivariate analyses revealed that age, tumor size, pathologic T stage, and N stage were significant risk factors associated with OS, CSS, and RFS in the training cohort (All *P*-values < 0.05) (Table S2-S5). The SII and PNI were independent predictors of OS, CSS, and RFS in the training cohort and validation cohort (Table S3). The significant influence of SII and PNI on OS, CSS, and RFS were further confirmed when running multivariate analysis for SII and PNI, respectively (Table S4-S5).

### The prognostic value of the combination of SII and PNI in patients with UTUC following RNU

**Figure 3:**
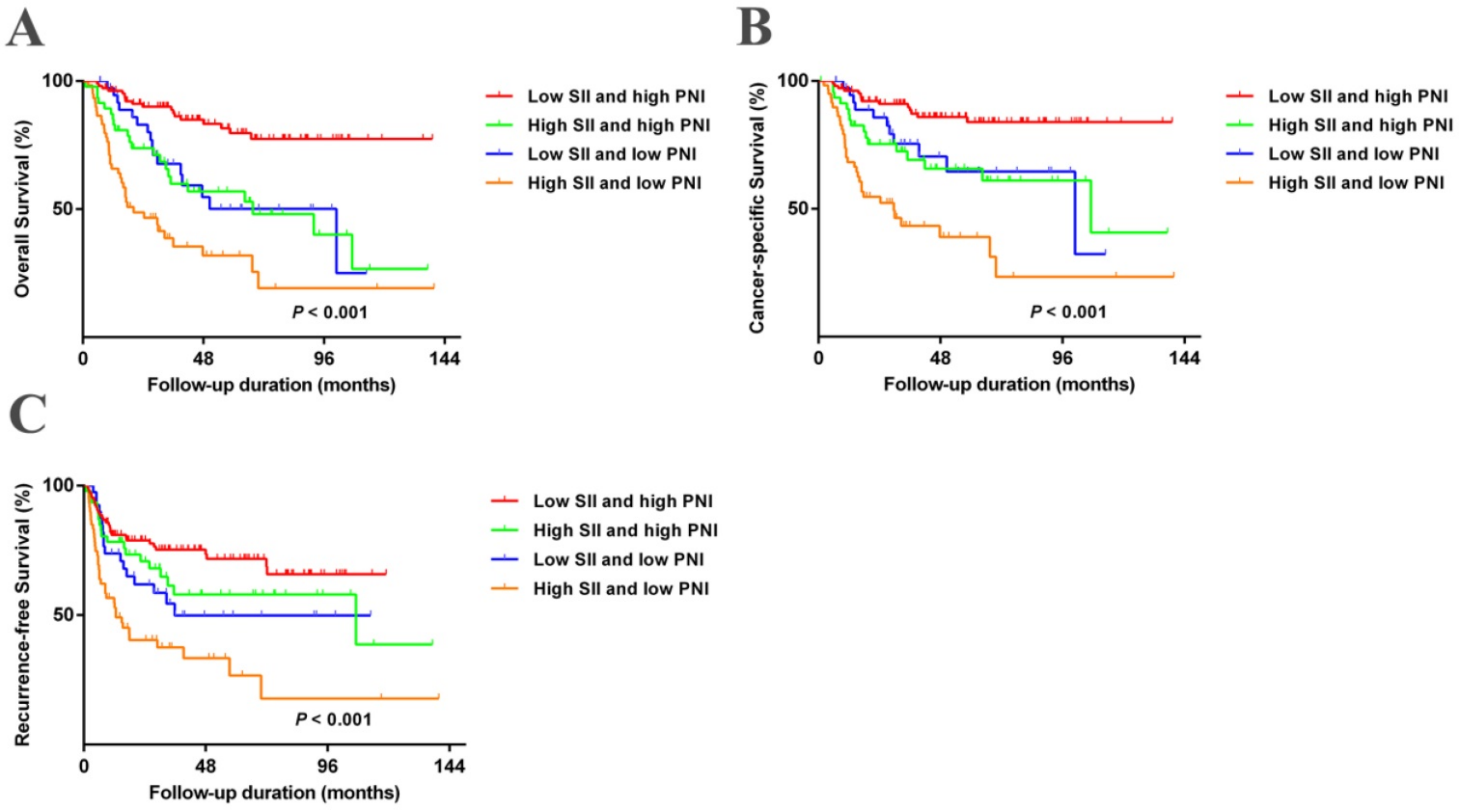
Kaplan-Meier analysis for OS, CSS, and RFS in patients with UTUC who was divided into 4 groups based on SII-PNI in the training cohort.

We further evaluated the predictive value of the combination of SII and PNI (SII-PNI). The patients were grouped as follows: low SII + high PNI, high SII + high PNI, low SII + low PNI, and high SII + low PNI. Figure 3 and Figure S2 show that patients with high SII + low PNI had the poorest OS, CSS, and RFS (All *P*-values < 0.05). A multivariate analysis was performed to investigate the effects of different SII and PNI combinations on OS, CSS, and RFS. Patients with high SII + low PNI had significantly worse OS, CSS, and RFS than those with low SII + high PNI in both the training cohort (OS: hazard ratio (HR) =3.853; 95% confidence interval (CI), 1.588-9.350; *P* = 0.003; CSS: HR = 5.197; 95%CI, 1.805-14.959; *P* = 0.002; RFS: HR = 2.915; 95%CI, 1.276-6.659; *P* = 0.011) and validation cohort (OS: HR =5.065; 95%CI, 1.798-14.269; *P* = 0.002; CSS: HR = 6.295; 95%CI, 1.864-21.265; *P* = 0.003; RFS: HR = 1.991; 95%CI, 1.814-4.872; *P* = 0.031) (Table 2). In addition, compared to SII or PNI alone and other inflammatory factors or clinical parameters, SII-PNI achieved the largest AUC (Figure 1 and Figure 7G), indicating that simultaneously high SII and low PNI values had better accurate predictive ability for predicting survival and could be identified as a prognostic staging tool for patients with UTUC.

### Establishment of nomograms and comparison of prognostic ability

**Figure 4:**
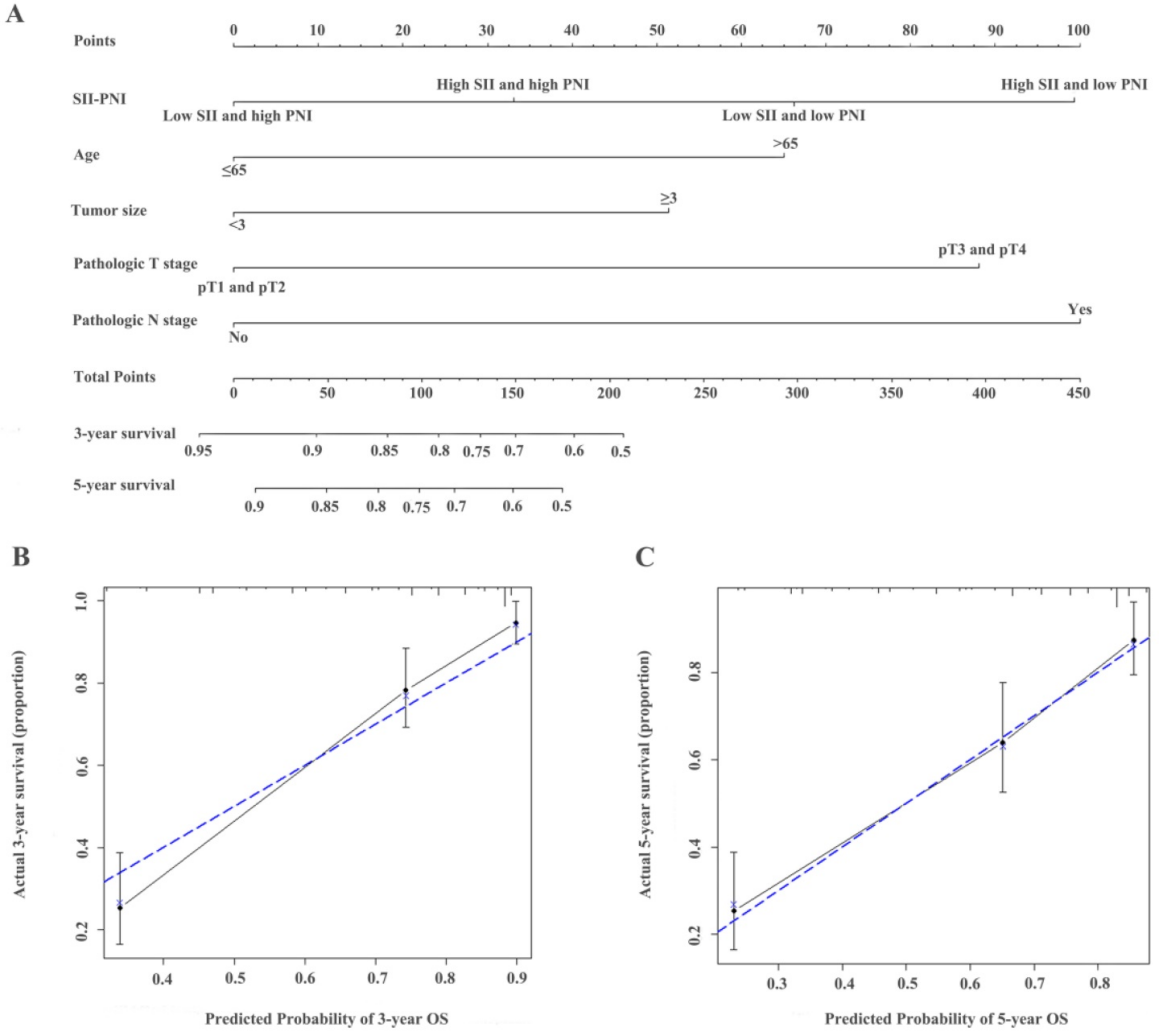
Construction of nomograms to predict OS (A) in patients with UTUC after surgery and Calibration curve for predicting 3- and 5-year survival of OS (B and C) in the training cohort.

**Figure 5:**
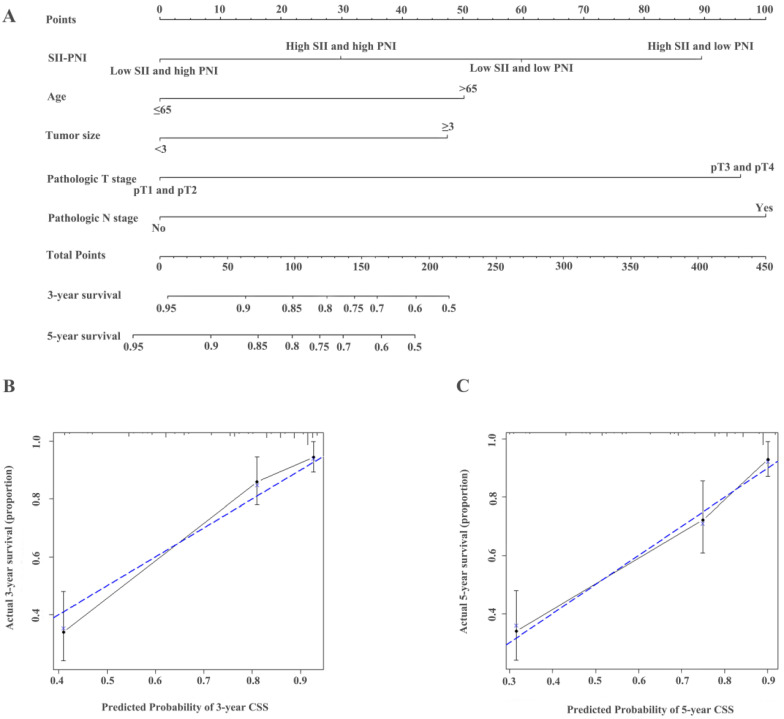
Construction of nomograms to predict CSS (**A**) in patients with UTUC after surgery and Calibration curve for predicting 3- and 5-year survival of CSS (**B and C**) in the training cohort.

**Figure 6:**
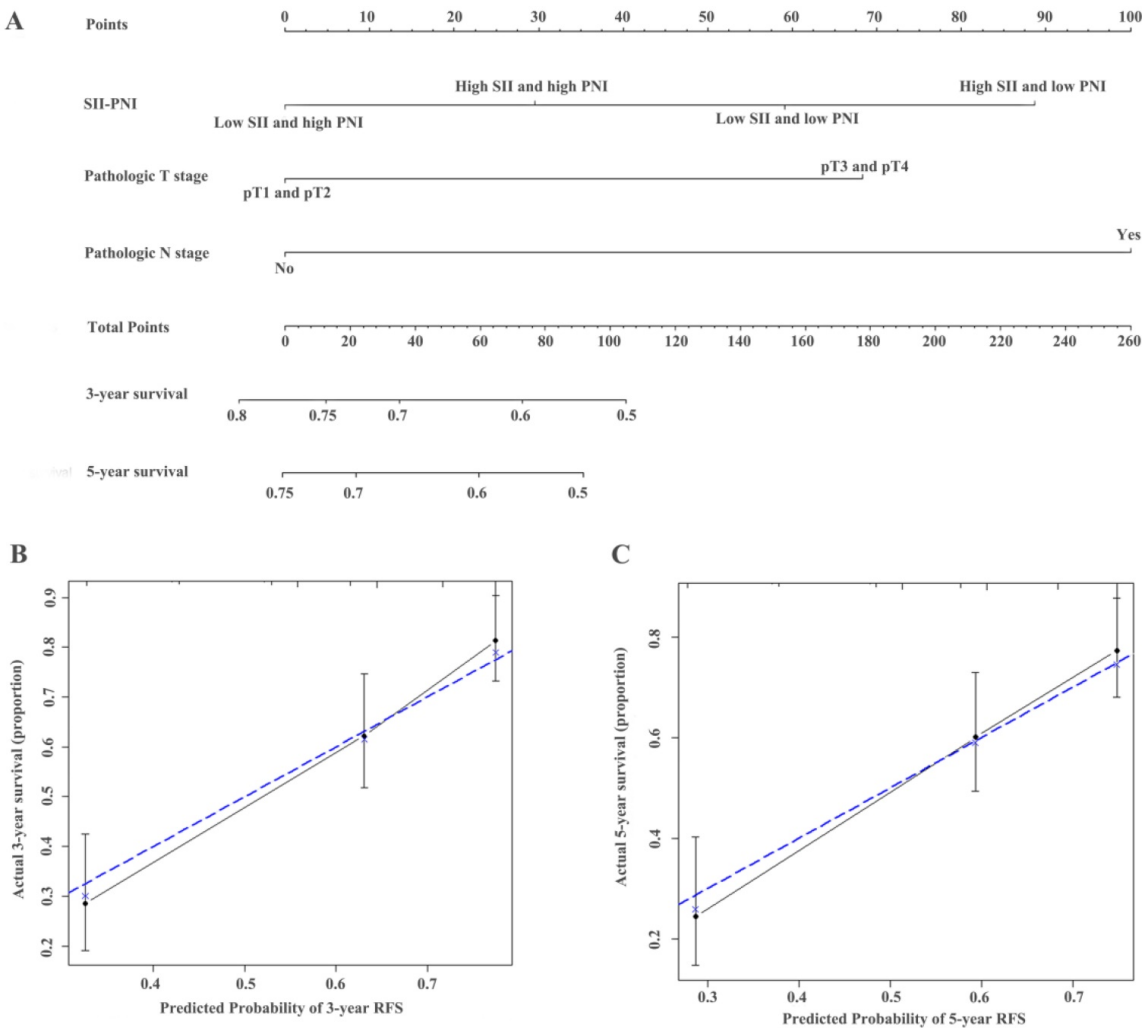
Construction of nomograms to predict RFS (**A**) in patients with UTUC after surgery and Calibration curve for predicting 3- and 5-year survival of RFS (**B and C**) in the training cohort.

The identified independent predictors from the multivariate analysis were used to construct nomograms for OS, CSS, and RFS (Figure 4A, Figure 5A, Figure 6A, Figure S3A, Figure S4A, and Figure S5A). The calibration plots of the three- and five- year survival probabilities showed that the predicted probability was highly consistent with the actual survival probability, indicating that the nomograms were well-calibrated (Figure 4B-C, Figure 5B-C, Figure 6B-C, Figure S3 B-C, Figure S4 B-C, and Figure S5 B-C).

**Figure 7:**
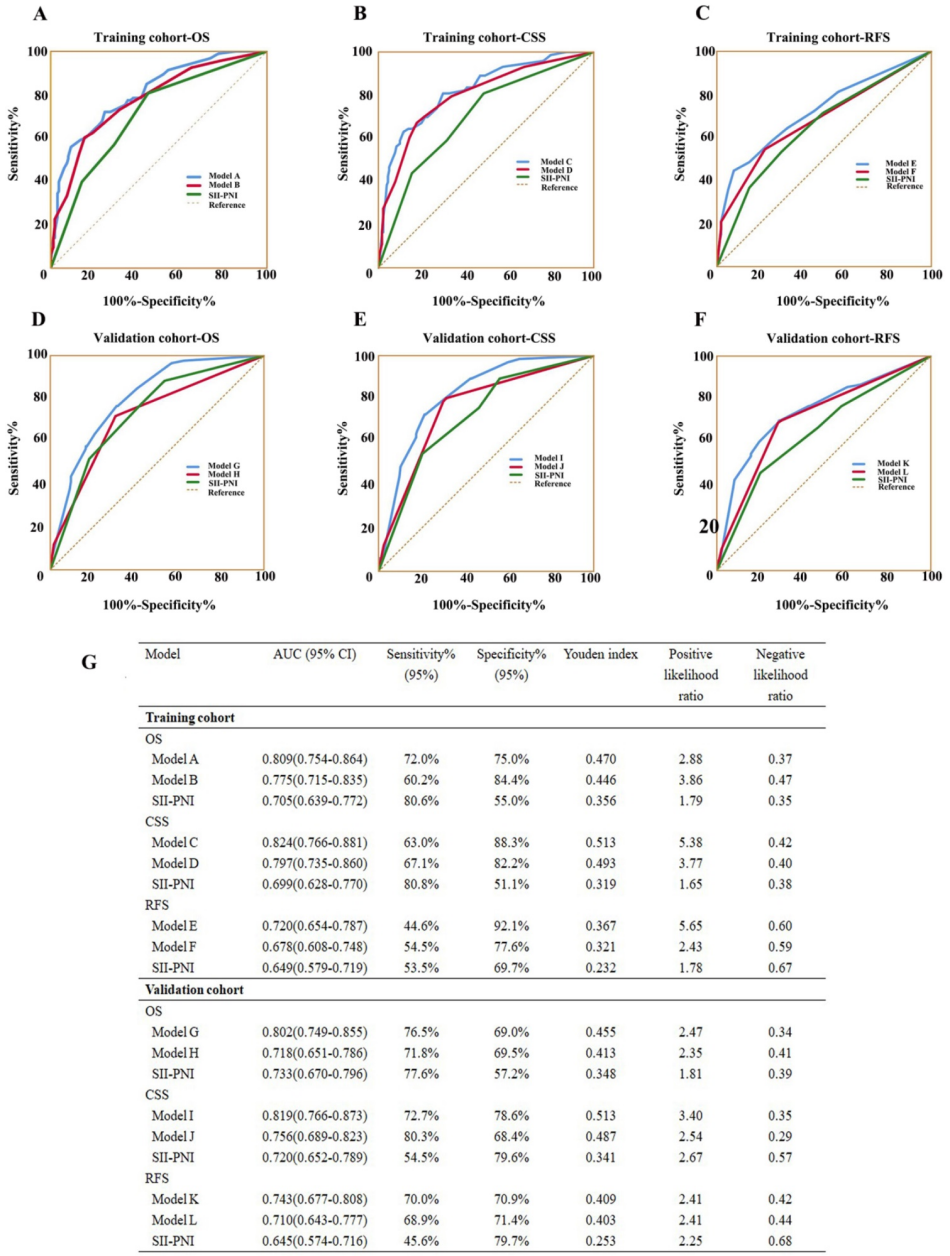
ROC analysis of the prognostic accuracy of SII-PNI for OS, CSS, and RFS in training cohort and validation cohort.

As shown in Table 3, the c-indexes for nomograms of OS, CSS, and RFS in the training cohort increased when SII-PNI was incorporated. SII-PNI combined with pathologic T stage, or N stage in the training cohort, respectively, had higher c-index values than those for SII or other parameters alone. In addition, by incorporating SII-PNI into developed models, the AUC and c-index of the nomograms increased in the training cohort (Table 3 and Figure 7). Moreover, these results were confirmed in the validation cohort (Table 3 and Figure 7). These findings indicated the ability of this new biomarker to improve the prognostic accuracy for patients with UTUC.

## Discussion

An accumulating number of studies have investigated the potential biomarkers to predict the prognosis of patients with UTUC to help urologists choose optimal treatments for individual patients. In the present study, a higher SII was independently associated with poorer prognosis in terms of shorter OS, CSS, and RFS, while a relatively higher PNI was associated with better OS, CSS, and RFS. The co-occurrence of higher SII and lower PNI was a strong independent prognostic factor of OS, CSS, and RFS, which identified patients with higher risk of mortality. In addition, with the largest AUC, the predictive ability of SII-PNI was superior to that of SII and PNI alone for predicting OS, CSS, and RFS. Most importantly, the predictive accuracy of an established nomogram was improved with increased c-index and AUC when incorporating SII-PNI. To our knowledge, this is the first report to investigate SII-PNI as a meaningful predictor for patients with UTUC.

Accumulating evidence indicates that increased systemic inflammatory response and a low nutritional status play important roles in the tumor progression and metastasis, which influence treatment outcomes, prognosis, and survival 12, 13. In recent years, the role of inflammation or nutrition-related factors, including NLR, PLR, albumin levels, and sarcopenia, in malignant tumors have attracted increased attention as significant predictors for various cancers that can reflect tumor' progression 7, 8, 14-16. In the current study, high SII and low PNI were correlated with old age, larger tumor size, LVI, pathologic T stage, N stage, and other clinical parameters indicative of an aggressive phenotype, which were consistent with the findings of a previous study. Our study results further demonstrated that elevated SII and decreased PNI were independent risk biomarkers in UTUC patients undergoing RNU.

The mechanisms of the complex interplay among systemic inflammatory response, nutritional status, tumor cell invasion, proliferation, and metastasis remain controversial. Several explanations have been proposed regarding the functions of circulating blood leukocytes, including neutrophils, platelets, and lymphocytes, which are the main contributors in the process of angiogenesis, invasion, and metastasis in the microenvironment of tumor-associated inflammation. First, infiltration of tumor-associated neutrophils may establish the tumor microenvironment by attracting inflammatory factors, including vascular endothelial growth factor, reactive oxygen species, and matrix metalloproteinase 9, which promote genetic instability and stimulate angiogenesis 17. In addition, neutrophils can inactive T cells and protect cancers cells from immune surveillance 18. Thus, increased neutrophil counts have tumor-promoting effects, contributing to tumor angiogenesis and metastasis 17, 19, 20. Second, it is reported that 10-57% patients with cancer are observed with thrombocytosis 9. Elevated platelets might induce epithelial-mesenchymal transition of circulating tumor cells 21, shield cancer cells from immune cells cytotoxicity and facilitate the extravasation of cancer cells 22-24, leading to the development and progression of cancer. Third, circulating lymphocytes have anti-tumor effects by secreting cytokines, including interferon (IFN)-γ and tumor necrosis factor (TNF)-α, and promoting cytotoxic cell death 20. The activation of the immune response, which can promote a protective response in patients with cancer, mainly depends on the level of lymphocytes, indicating their powerful antitumor properties. In addition, a previous study reported that a high SII was associated with elevated serum levels of inflammatory cytokines and chemokines 25. Therefore, a high SII level may indicate a significant inflammatory response with increased participation of neutrophils and platelets; increased cytokine and growth factor expression and low immune response with decreased infiltration of lymphocytes. As a result, survival after surgery is poor in patients with increased SII.

Malnutrition can suppress immune system functions, increasing patient vulnerability to infection or cancer via cell-mediated mechanisms and other immune pathways 26, 27. Malnutrition can also delay patients' surgery or adjuvant therapy. Thus, malnutrition is generally associated with unfavorable outcomes after surgery in many solid malignancies 9, 12. However, few studies have investigated the association between nutritional status and systemic inflammation in patients with UTUC. PNI, a combination of serum albumin level and lymphocyte count, has been used to assess the nutritional status and has been identified as a risk factor for prognosis in UTUC patients. In the current study, serum PNI level was significantly negatively related to SII, NLR, PLR, and MLR. Thus, to some extent, we assume that a worse nutritional status in patients with cancer also indicates impairment of immune response and excessive infiltration of inflammatory components, leading to tumor development. The detailed mechanisms required further validation in the future studies.

Our results demonstrated that both SII and PNI were independent predictors of OS, CSS, and RFS in patients with UTUC. However, the predictive ability of the two biomarkers was relatively low with AUC of 0.647 (0.577-0.717) for SII and 0.596 (0.523-0.669) for PNI. The combination of SII and PNI had a better prognostic value over SII or PNI alone, with an AUC of 0.705 (0.639-0.772). The predictive value of SII-PNI was also validated in an independent cohort.

This study has several limitations. First, this was a retrospective study; thus, there may be selection bias during patient enrollment and data collection. However, another independent cohort was used to validate the results from the training cohort. Second, we did not assess other inflammatory scores, including C-reactive protein, IFN-γ and TNF-α levels because of incomplete data. Third, the role of dynamic changes in SII-PNI on survival has not been evaluated because we lacked related data. Further studies should include this important information.

## Conclusions

The findings of the current study suggest that the combination of SII and PNI may be a simple, non-invasive, easily accessible, and potentially effective indicator to evaluate the prognosis of patients of UTUC. Urologists should consider this novel biomarker for clinical decision-making and risk stratification.

## Supplementary Material

Supplementary figures and tables.

## Figures and Tables

**Table 1:** Characteristics of training and validation cohorts according to SII or PNI

Variable	Training cohort (n = 253)						Validation cohort (n = 272)					
	SII			PNI			SII			PNI		
	≥672.44 (n = 107)	< 672.44 (n = 146)	*P*-value	≥47.83 (n = 153)	< 47.83 (n = 100)	*P*-value	≥672.44 (n = 107)	< 672.44 (n = 165)	*P*-value	≥47.83 (n = 126)	< 47.83 (n = 146)	*P*-value
Age (>65 vs ≤65 years)	69/38	92/54	0.810	86/67	75/25	0.002*	66/41	92/73	0.333	59/67	99/47	<0.001*
Gender (Male vs Female)	81/26	99/47	0.171	107/46	73/27	0.599	79/28	103/62	0.051	87/39	95/51	0.487
ASA grade (≥3 vs <3)	27/80	34/112	0.721	28/125	33/67	0.008*	22/85	23/142	0.151	16/110	29/117	0.113
BMI (≥25 vs <25, Kg/m^2^)	10/97	38/108	0.001*	39/114	9/91	0.001*	30/77	61/104	0.127	44/82	47/99	0.634
Hydronephrosis (Yes vs No)	74/33	94/52	0.427	97/56	71/29	0.211	77/30	110/55	0.357	75/51	112/34	0.002*
Surgical approach (laparoscopic vs open)	28/79	57/89	0.032*	55/98	30/70	0.327	91/16	154/11	0.026*	119/7	126/20	0.025*
NLR, Mean ± SD	5.22 ± 3.79	2.14 ± 0.83	<0.001*	2.62 ± 1.43	4.70 ± 4.06	<0.001*	5.02 ± 2.86	2.03 ± 0.81	<0.001*	3.98 ± 2.81	2.32 ± 1.33	<0.001*
PLR, Mean ± SD	216.49 ± 110.38	109.59 ± 32.75	<0.001*	124.43 ± 50.51	201.28 ± 119.24	<0.001*	184.34 ± 78.49	110.01 ± 36.07	<0.001*	164.17 ± 75.16	110.38 ± 41.00	<0.001*
MLR, Mean ± SD	0.52 ± 0.36	0.26 ± 0.10	<0.001*	0.28 ± 0.13	0.51 ± 0.37	<0.001*	0.46 ± 0.28	0.26 ± 0.12	<0.001*	0.41 ± 0.26	0.25 ± 0.11	<0.001*
Anemia (Yes vs No)	61/46	46/61	<0.001*	43/110	64/36	**<0.001**	44/63	36/129	0.001*	24/102	56/90	<0.001*
Hypoproteinemia (Yes vs No)	16/91	6/140	0.002*	1/152	21/79	**<0.001**	13/94	10/155	0.078	0/126	23/123	<0.001*
**CKD stage**			0.041*			<0.001*			0.183			0.677
CKD 1	8	19		25	2		29	35		31	33	
CKD 2	30	56		57	29		39	51		37	53	
CKD 3	53	61		64	50		32	71		51	52	
CKD 4	11	9		6	14		7	8		7	8	
CKD 5	5	1		1	5		0	0		0	0	
Tumor size (≥3 vs <3, cm)	46/61	50/96	0.157	55/98	41/59	0.418	46/61	50/115	0.032*	30/96	66/80	<0.001*
**Tumor site**			0.677			0.246			0.052			0.847
Pelvicalyceal	69	94		100	63		60	89		71	78	
Ureter	32	47		49	30		36	70		48	58	
Both	6	5		4	7		11	6		7	10	
Multifocality(Yes vs No)	27/80	21/125	0.030*	25/128	23/77	0.187	25/82	40/125	0.868	26/100	39/107	0.241
**Pathologic T stage**			<0.001*			<0.001*			<0.001*			0.017*
≥pT3	53	54		39	47		62	52		41	73	
< pT3	33	113		114	53		45	113		85	73	
N stage (N1 vs N0)	20/87	4/142	<0.001*	10/143	14/86	0.048*	12/95	5/160	0.006*	6/120	11/135	0.346
Tumor grade (≥3 vs <3)	87/20	108/38	0.170	116/37	79/21	0.556	84/23	113/52	0.071	84/42	113/33	0.048*
LVI (Yes vs No)	28/79	13/133	<0.001*	16/137	25/75	0.002*	24/83	14/151	0.001*	17-109	21/125	0.833
Adjuvant therapy (Yes vs No)	18/89	10/136	0.012*	17/136	11/89	0.978	35/72	39/126	0.100	36/90	38/108	0.638
Follow-up duration, months, median (IQR)	20.30 (10.90-46.60)	42.70 (24.35-69.48)	<0.001*	43.80 (22.75-70.70)	24.45 (10.90-39.68)	<0.001*	32.90 (16.90-52.00)	52.20 (33.60-73.50)	<0.001*	51.65 (31.85-66.05)	37.10 (24.00-64.08)	<0.001*
All-cause death, n (%)	59 (55.14%)	34 (23.29%)	<0.001*	40 (25.16%)	53 (53.00%)	<0.001*	53 (49.53%)	32 (19.39%)	<0.001*	19 (15.08%)	66 (45.21%)	<0.001*
Cancer-specific death, n (%)	48 (44.86%)	25 (17.12%)	<0.001*	30 (18.87%)	43 (43.00%)	<0.001*	45 (42.6%)	21 (12.73%)	<0.001*	16 (12.70%)	50 (34.25%)	<0.001*
Recurrence after surgery, n (%)	55 (51.40%)	46 (31.51%)	<0.001*	47 (30.72%)	54 (54.00%)	**<0.001**	50 (46.73%)	40 (24.24%)	<0.001*	30 (23.81%)	60 (41.10%)	0.003*

Note: *statistically significant; Abbreviations: ASA; American Society of Anesthesiologists; BMI, body mass index; SII, systemic immune-inflammation index; PNI, prognostic nutritional index; NLR, neutrophil-to-lymphocyte ratio; PLR, platelet-to-lymphocyte ratio; MLR, monocyte-to-lymphocyte ratio; CKD, chronic kidney disease; LVI, lymphovascular invasion; IQR, interquartile range.

**Table 2:** Multivariate analysis of variables for the prediction of survival outcomes in training and validation cohorts when SII-PNI was incorporated

Variables	Overall survival			Cancer-specific survival			Recurrence-free survival		
	HR	95%CI	*P* value	HR	95%CI	*P* value	HR	95%CI	*P* value
**Training cohort**									
Age (>65 vs ≤65 years)	2.086	1.025-5.100	0.043*	1.811	1.055-3.107	0.031*	1.536	0.991-2.381	0.055
SII-PNI									
Low SII+high PNI	1.000	Reference	1.000	1.000	Reference	1.000	1.000	Reference	1.000
High SII+high PNI vs Low SII+high PNI	2.481	1.058-5.817	0.037*	3.011	1.103-8.224	0.032*	1.626	0.722-3.662	0.241
Low SII+low PNI vs Low SII+high PNI	2.529	1.205-5.310	0.014*	2.390	0.998-5.723	0.050	1.810	0.948-3.456	0.072
High SII+low PNI vs Low SII+high PNI	3.853	1.588-9.350	0.003*	5.197	1.805-14.959	0.002*	2.915	1.276-6.659	0.011*
NLR (≥2.53 vs<2.53)	0.770	0.382-1.550	0.464	0.502	0.217-1.160	0.107	0.555	0.284-1.083	0.084
PLR (≥126.88 vs<126.88)	1.373	0.767-2.456	0.286	1.332	0.691-2.566	0.392	1.275	0.746-2.177	0.375
MLR (≥0.35 vs<0.35)	0.797	0.484-1.310	0.371	0.804	0.455-1.422	0.454	0.934	0.571-1.526	0.784
Tumor size (≥3 vs<3)	1.722	1.117-2.654	0.014*	1.751	1.073-2.857	0.025*	1.471	0.977-2.216	0.065
Pathologic T stage (≥pT3 vs < pT3)	2.476	1.498-4.092	<0.001*	2.837	1.578-5.100	<0.001*	1.686	1.029-2.764	0.038*
N stage (N1 vs N0)	2.286	1.025-5.100	0.043*	2.062	1.719-5.919	0.048*	2.285	1.022-5.105	0.044*
Tumor grade (≥3 vs <3)	1.355	0.630-2.918	0.437	2.062	0.719-5.919	0.178	1.560	0.822-2.959	0.174
LVI (Yes vs No)	1.580	0.759-3.290	0.222	2.071	0.958-4.477	0.064	1.333	0.644-2.758	0.439
**Validation cohort**									
Age (>65 vs ≤65 years)	1.056	0.647-1.724	0.828	0.876	0.511-1.500	0.629	0.984	0.621-1.558	0.945
SII-PNI									
Low SII+high PNI	1.000	Reference	1.000	1.000	Reference	1.000	1.000	Reference	1.000
High SII+high PNI vs Low SII+high PNI	2.963	0.990-8.865	0.052	3.867	1.143-13.085	0.030*	0.982	0.365-2.644	0.672
Low SII+low PNI vs Low SII+high PNI	2.791	1.266-6.151	0.011*	2.464	0.947-6.414	0.065	1.059	0.537-2.086	0.369
High SII+low PNI vs Low SII+high PNI	5.065	1.798-14.269	0.002*	6.295	1.864-21.265	0.003*	1.991	1.814-4.872	0.031*
NLR (≥2.53 vs<2.53)	0.592	0.270-1.300	0.191	0.580	0.231-1.453	0.245	0.931	0.444-1.951	0.850
PLR (≥126.88 vs<126.88)	1.517	0.846-2.720	0.162	1.374	0.734-2.575	0.321	1.301	0.731-2.31	0.370
MLR (≥0.35 vs<0.35)	1.561	0.907-2.686	0.108	1.439	0.792-2.614	0.232	1.290	0.770-2.161	0.333
Tumor size (≥3 vs<3)	1.107	0.698-1.756	0.666	1.140	0.676-1.924	0.623	1.112	0.709-1.744	0.644
Pathologic T stage (≥pT3 vs < pT3)	3.000	1.767-5.092	<0.001*	4.621	2.396-8.911	<0.001*	3.308	1.993-5.491	<0.001*
N stage (N1 vs N0)	2.070	1.200-5.228	0.027*	1.831	1.085-4.891	0.038*	1.304	1.010-3.153	0.045*
Tumor grade (≥3 vs <3)	1.763	0.896-3.467	0.101	1.757	0.768-4.019	0.182	1.607	0.844-3.058	0.149
LVI (Yes vs No)	1.047	0.472-2.327	0.909	1.090	0.484-2.456	0.835	1.332	0.664-2.672	0.420

Note: *statistically significant.

**Table 3:** C-index analysis of the prognostic accuracy of SII-PNI and other variables for OS, CSS, and RFS in training cohort and validation cohort

Characteristics	OS	CSS	RFS
**Training cohort**			
Age	0.566 (0.515-0.617)	0.551 (0.493-0.609)	-
SII-PNI	0.701 (0.648-0.754)	0.698 (0.638-0.758)	0.638 (0.582-0.694)
Tumor size	0.567 (0.514-0.620)	0.567 (0.507-0.627)	-
Pathologic T stage	0.689 (0.641-0.737)	0.713 (0.658-0.765)	0.633 (0.585-0.681)
N stage	0.619 (0.574-0.664)	0.639 (0.587-0.691)	0.591 (0.553-0.629)
SII-PNI + Pathologic T stage	0.760 (0.710-0.810)	0.777 (0.722-0.832)	0.680 (0.626-0.734)
SII-PNI + N stage	0.765 (0.717-0.813)	0.778 (0.725-0.831)	0.688 (0.632-0.744)
Model A	0.804 (0.761-0.847)	-	-
Model B	0.780 (0.734-0.826)	-	-
Model C	-	0.817 (0.771-0.863)	-
Model D	-	0.802 (0.752-0.852)	-
Model E	-	-	0.707 (0.654-0.760)
Model F	-	-	0.669 (0.619-0.719)
**Validation cohort**			
SII-PNI	0.698 (0.644-0.752)	0.706 (0.647-0.765)	0.592 (0.526-0.658)
Pathologic T stage	0.697 (0.648-0.746)	0.727 (0.677-0.777)	0.642 (0.584-0.700)
N stage	0.561 (0.521-0.601)	0.555 (0.511-0.599)	0.529 (0.492-0.566)
SII-PNI + Pathologic T stage	0.774 (0.729-0.819)	0.798 (0.750-0.846)	0.663 (0.597-0.729)
SII-PNI + N stage	0.722 (0.669-0.775)	0.721 (0.663-0.779)	0.612 (0.547-0.677)
Model G	0.783 (0.737-0.829)	-	-
Model H	0.719 (0.668-0.770)	-	-
Model I	-	0.803 (0.755-0.851)	-
Model J	-	0.745 (0.694-0.796)	-
Model K	-	-	0.670 (0.604-0.736)
Model L	-	-	0.651 (0.590-0.712)

Model A (for OS) = age + SII-PNI + tumor size + pathologic T stage + N stage; Model B (for OS) = age + tumor size + pathologic T stage + N stage; Model C (for CSS) = age + SII-PNI + tumor size + pathologic T stage + N stage; Model D (for CSS) = age + tumor size + pathologic T stage + N stage; Model E (for RFS) = SII-PNI + pathologic T stage + N stage; Model F (for RFS) = pathologic T stage + N stage; Model G (for OS) = SII-PNI + pathologic T stage + N stage; Model H (for OS) = pathologic T stage + N stage; Model I (for CSS) = SII-PNI + pathologic T stage + N stage; Model J (for CSS) = pathologic T stage + N stage; Model K (for RFS) = SII-PNI + pathologic T stage + N stage; Model L (for RFS) = pathologic T stage + N stage.

## Abbreviations

SII: systemic immune-inflammation index
PNI: prognostic nutritional index
UTUC: upper tract urothelial carcinoma
RNU: radical nephronureterectomy
OS: overall survival
CSS: cancer-specific survival
RFS: recurrence-free survival
AUC: area under the curve
NLR: neutrophil-to-lymphocyte ratio
PLR: platelet-to-lymphocyte ratio
MLR: monocyte-to-lymphocyte ratio
c-index: concordance index
IQR: interquartile range
ASA: American Society of Anesthesiologists
BMI: body mass index
CKD: chronic kidney disease
LVI: lymphovascular invasion
HR: hazard ratio
CI: confidence interval
IFN-γ: interferon -γ
TNF-α: tumor necrosis factor-α
