# Supplementary material for: Association of preoperative systemic Immune-inflammation Index and Prognostic Nutritional Index with survival in patients with Upper Tract Urothelial Carcinoma
Source: J Cancer. 2020 Jul 25;11(19):5665–77. doi: 10.7150/jca.44915 (PMC7477451; doi:10.7150/jca.44915)
Supplement: Supplementary file 1 — Supplementary figures and tables. [file jcav11p5665s1.pdf]

Supplementary materials

Supplementary 1.

Table S1 Overall characteristics of training and validation cohorts

Variable	Training cohort (n = 253)	Validation cohort (n = 272)	*P* value
Age, years, Mean ± SD	67.59 ± 10.49	65.87 ± 10.35	0.059
Gender (Male vs Female)	180/73	182/90	0.295
ASA grade			0.496
1	21	16	
2	195	211	
3	37	45	
BMI (≥25 vs <25, Kg/m^2)	22.47 ± 2.86	23.86 ± 3.25	<0.001*
Hydronephrosis (Yes vs No)	168/85	187/85	0.566
Surgical approach (laparoscopic vs open)	85/168	245/27	<0.001*
NLR, Mean ± SD	3.44 ±2.96	3.21 ± 2.40	0.315
PLR, Mean ± SD	154.80 ± 92.42	139.25 ± 67.23	0.127
MLR, Mean ± SD	0.37 ± 0.28	0.34 ± 0.22	0.101
SII, Mean ± SD	770.21 ± 640.00	685.81 ± 590.87	0.117
PNI, Mean ± SD	49.38 ± 6.00	47.87 ± 6.22	0.135
Anemia (Yes vs No)	107/146	80/192	0.002*
Hypoproteinemia (Yes vs No)	22/231	23/249	0.922
CKD stage			<0.001*
CKD 1	27	64	
CKD 2	86	90	
CKD 3	114	103	
CKD 4	20	15	
CKD 5	6	0	
Tumor size (≥3 vs<3, cm)	96/157	96/176	0.529
Tumor site			0.075
Pelvicalyceal	163	149	
Ureter	79	106	
Both	11	17	
Multifocality (Yes vs No)	48/205	65/207	0.170
Pathologic T stage (≥pT3 vs < pT3)	88/165	114/158	0.076
N stage (N1 vs N0)	24/229	17/255	0.167
Tumor grade (≥3 vs <3)	195/58	197/75	0.221
LVI (Yes vs No)	41/212	38/234	0.474
Adjuvant therapy (Yes vs No)	28/225	74/198	<0.001*

Note: *statistically significant

Supplementary 2.

Table S2 Univariate analysis of variables for the prediction of survival outcomes in training and validation cohorts

Variables	Overall survival			Cancer-specific survival			Recurrence-free survival		
	HR	95%CI	*P* value	HR	95%CI	*P* value	HR	95%CI	*P* value
**Training cohort**									
Gender (Male vs Female)	0.890	0.571-1.386	0.606	0.896	0.543-1.477	0.666	1.071	0.693-1.656	0.757
Age (>65 vs ≤65 years)	2.018	1.272-3.204	**0.003***	1.774	1.065-2.955	**0.028***	1.514	0.994-2.307	0.053
BMI (≥25 vs <25)	0.418	0.210-0.832	**0.013***	0.421	0.193-0.918	**0.030***	0.444	0.237-0.832	0.011*
ASA grade (≥3 vs <3)	1.527	0.984-2.370	0.059	1.342	0.808-2.229	0.256	1.131	0.724-1.767	0.589
Hydronephrosis (Yes vs No)	1.542	0.969-2.454	0.068	1.892	1.086-3.295	**0.024***	1.989	1.240-3.191	0.004*
Surgical approach (laparoscopic vs open)	0.702	0.435-1.135	0.149	0.728	0.427-1.242	0.245	0.722	0.461-1.129	0.153
SII (≥672.44 vs<672.44)	3.348	2.191-5.118	**<0.001***	3.580	2.203-5.818	**<0.001***	2.069	1.396-3.064	<0.001*
NLR (≥2.53 vs<2.53)	2.684	1.704-4.228	**<0.001***	2.351	1.424-3.882	**0.001***	1.523	1.019-2.276	0.040*
PLR (≥126.88 vs<126.88)	2.786	1.784-4.350	**<0.001***	2.680	1.622-4.430	**<0.001***	1.852	1.236-2.775	0.003*
MLR (≥0.35 vs<0.35)	2.216	1.471-3.338	**<0.001***	2.215	1.395-3.517	**0.001***	1.718	1.160-2.545	0.007*
PNI (<47.83 vs≥47.83)	3.003	1.984-4.547	**<0.001***	3.116	1.947-4.987	**<0.001**	2.411	1.624-3.578	<0.001*
Anemia (Yes vs No)	1.903	1.263-2.867	**0.002***	1.792	1.130-2.843	0.013*	1.767	1.194-2.614	0.004*
Hypoproteinemia (Yes vs No)	2.596	1.464-4605	**0.001***	2.736	1.467-5.106	**0.002***	1.871	1.043-3.357	0.036*
CKD stage									
CKD 1	1.000	Reference	1.000	1.000	Reference	1.000	1.000	Reference	1.000
CKD 2-3	2.877	1.050-7.882	**0.040***	2.919	0.913-9.331	0.071	2.461	0.996-6.080	0.051
CKD 4-5	6.075	2.019-18.283	**0.001***	6.138	1.740-21.659	**0.005***	4.805	1.753-13.170	0.002*
Tumor size (≥3 vs<3)	1.584	1.050-2.388	**0.028***	1.629	1.026-2.587	0.039*	1.526	1.030-2.262	0.035*
Tumor site									
Pelvicalyceal	1.000	Reference	1.000	1.000	Reference	1.000	1.000	Reference	1.000
Ureter	1.421	0.920-2.194	0.113	1.627	1.000-2.645	0.050	1.621	1.072-2.451	0.022*
Both	1.695	0.677-4.244	0.260	2.335	0.919-5.935	0.075	2.213	1.007-4.861	0.048*
Multifocality (Yes vs No)	1.652	1.031-2.649	**0.037***	1.764	1.045-2.978	**0.034***	1.402	0.880-2.233	0.155
Pathologic T stage (≥pT3 vs < pT3)	4.445	2.921-6.766	<0.001*	5.627	3.447-9.187	<0.001*	2.978	2.004-4.426	<0.001*
N stage (N1 vs N0)	7.410	4.462-12.306	**<0.001***	8.855	5.180-15.138	**<0.001***	5.186	3.174-8.471	<0.001*
Tumor grade (≥3 vs <3)	2.638	1.275-5.456	**0.009***	4.435	1.616-12.168	**0.004***	2.251	1.231-4.118	0.008*
LVI (Yes vs No)	4.789	3.089-7.425	**<0.001***	6.372	3.967-10.235	**<0.001***	3.774	2.459-5.792	<0.001*
Adjuvant therapy (Yes vs No)	2.820	1.732-4.590	**<0.001***	3.584	2.140-6.003	**<0.001***	3.476	2.171-5.565	<0.001*
**Validation cohort**									
Gender (Male vs Female)	1.421	0.880-2.293	0.150	1.536	0.885-2.668	0.127	1.269	0.804-2.002	0.306
Age (>65 vs ≤65 years)	1.347	0.859-2.111	0.195	1.111	0.675-1.827	0.680	1.087	0.711-1.660	0.701
BMI (≥25 vs <25)	0.766	0.477-1.230	0.269	0.742	0.432-1.276	0.281	0.832	0.530-1.306	0.424

ASA grade (≥3 vs <3)	1.089	0.623-1.903	0.765	0.807	0.399-1.631	0.551	0.934	0.528-1.653	0.815
Hydronephrosis (Yes vs No)	1.328	0.822-2.144	0.246	1.525	0.868-2.678	0.142	1.233	0.781-1.945	0.369
Surgical approach (laparoscopic vs open)	0.386	0.224-0.665	**0.001***	0.390	0.208-0.730	**0.003***	0.438	0.247-0.774	0.005*
SII (≥672.44 vs<672.44)	3.433	2.208-5.338	**<0.001***	4.379	2.603-7.368	**<0.001***	2.448	1.612-3.717	<0.001*
NLR (≥2.53 vs<2.53)	2.700	1.699-4.290	**<0.001***	3.102	1.802-5.339	**<0.001***	2.166	1.400-3.350	0.001*
PLR (≥126.88 vs<126.88)	3.418	2.179-5.361	**<0.001***	3.434	2.073-5.688	**<0.001***	2.413	1.527-3.815	<0.001*
MLR (≥0.35 vs<0.35)	2.672	1.742-4.098	**<0.001***	2.572	1.584-4.177	**<0.001***	2.045	1.346-3.106	0.001*
PNI (<47.83 vs≥47.83)	3.611	2.162-6.029	**<0.001***	3.154	1.795-5.540	**<0.001***	2.011	1.297-3.119	0.002*
Anemia (Yes vs No)	2.319	1.513-3.555	**<0.001***	2.008	1.232-3.274	**0.005***	1.852	1.211-2.831	0.004*
Hypoproteinemia (Yes vs No)	2.015	1.067-3.806	**0.031***	1.897	0.904-3.978	0.090	1.238	0.599-2.560	0.564
CKD stage									
CKD 1	1.000	Reference	1.000	1.000	Reference	1.000	1.000	Reference	1.000
CKD 2-3	0.867	0.517-1.455	0.590	0.698	0.404-1.206	0.197	0.760	0.474-1.216	0.252
CKD 4-5	1.016	0.405-2.549	0.973	0.539	0.158-1.832	0.322	0.413	0.124-1.375	0.150
Tumor size (≥3 vs<3)	1.640	1.064-2.527	**0.025***	1.797	1.106-2.921	**0.018***	1.473	0.965-2.247	0.073
Tumor site									
Pelvicalyceal	1.000	Reference	1.000	1.000	Reference	1.000	1.000	Reference	1.000
Ureter	0.754	0.472-1.204	0.237	0.766	0.452-1.300	0.323	0.814	0.523-1.266	0.361
Both	2.258	1.138-4.480	**0.020***	1.944	0.866-4.365	0.107	1.390	0.630-3.065	0.414
Multifocality (Yes vs No)	1.495	0.942-2.374	0.088	1.390	0.815-2.369	0.226	1.159	0.722-1.861	0.542
Pathologic T stage (≥pT3 vs < pT3)	4.964	3.101-7.945	<0.001*	7.596	4.189-13.774	<0.001*	4.543	2.892-7.138	**<0.001***
N stage (N1 vs N0)	4.368	2.356-8.099	**<0.001***	4.051	1.997-8.218	**<0.001***	2.866	1.481-5.545	0.002*
Tumor grade (≥3 vs <3)	3.275	1.794-5.978	**<0.001***	3.862	1.835-8.128	**<0.001***	2.941	1.655-5.225	<0.001*
LVI (Yes vs No)	2.941	1.766-4.898	**<0.001***	3.108	1.782-5.423	**<0.001***	2.629	1.607-4.300	<0.001*
Adjuvant therapy (Yes vs No)	1.655	1.059-2.587	**0.027***	1.991	1.213-3.267	**0.006***	1.609	1.041-2.487	0.032*

Note: *statistically significant

Supplementary 3.

Table S3 Multivariate analysis of variables for the prediction of survival outcomes in training and validation cohorts when including SII and PNI

Variables	Overall survival			Cancer-specific survival			Recurrence-free survival		
	HR	95%CI	*P* value	HR	95%CI	*P* value	HR	95%CI	*P* value
**Training cohort**									
Age (>65 vs ≤65 years)	2.094	1.289-3.402	**0.003***	1.820	1.062-3.122	**0.029***	1.536	0.991-2.380	0.055
SII (≥672.44 vs<672.44)	1.861	0.960-3.607	**0.046***	2.511	1.109-5.688	**0.027***	1.618	0.832-3.143	**0.045***
NLR (≥2.53 vs<2.53)	0.812	0.401-1.644	0.562	0.510	0.217-1.195	0.121	0.555	0.285-1.080	0.083
PLR (≥126.88 vs<126.88)	1.409	0.780-2.544	0.256	1.366	0.706-2.644	0.355	1.275	0.747-2.177	0.373
MLR (≥0.35 vs<0.35)	0.796	0.482-1.316	0.374	0.805	0.453-1.428	0.458	0.933	0.572-1.523	0.782

PNI (<47.83 vs≥47.83)	1.858	1.155-2.900	**0.011***	1.937	1.116-3.364	**0.019***	1.801	1.143-2.837	**0.011***
Tumor size (≥3 vs<3)	1.684	1.095-2.588	**0.018***	1.721	1.058-2.797	**0.029***	1.470	0.980-2.205	0.062
Pathologic T stage (≥pT3 vs < pT3)	2.561	1.557-4.213	**<0.001***	2.910	1.629-5.196	**<0.001***	1.688	1.034-2.754	**0.036***
N stage (N1 vs N0)	2.222	1.996-4.959	**0.011***	1.911	1.036-4.370	**0.025***	2.282	1.027-5.070	**0.043***
Tumor grade (≥3 vs <3)	1.413	0.660-3.025	0.374	2.124	0.744-6.063	0.159	1.561	0.824-2.956	0.172
LVI (Yes vs No)	1.505	0.729-3.107	0.269	2.009	0.938-4.302	0.073	1.332	0.645-2.749	0.438
**Validation cohort**									
Age (>65 vs ≤65 years)	1.039	0.638-1.694	0.877	0.863	0.505-1.474	0.589	1.001	0.634-1.582	0.996
SII (≥672.44 vs<672.44)	2.054	0.977-4.321	**0.048***	2.879	1.219-6.796	**0.016***	1.537	0.783-3.019	**0.042***
NLR (≥2.53 vs<2.53)	0.604	0.271-1.342	0.216	0.588	0.230-1.502	0.267	0.872	0.427-1.781	0.707
PLR (≥126.88 vs<126.88)	1.491	0.831-2.675	0.181	1.360	0.725-2.551	0.338	1.343	0.758-2.380	0.313
MLR (≥0.35 vs<0.35)	1.513	0.883-2.594	0.132	1.400	0.775-2.528	0.265	1.359	0.817-2.260	0.237
PNI (<47.83 vs≥47.83)	2.234	1.227-4.067	**0.009***	1.975	0.998-3.908	**0.031***	1.397	0.831-2.347	**0.037***
Tumor size (≥3 vs<3)	1.092	0.687-1.735	0.710	1.126	0.667-1.900	0.657	1.131	0.723-1.770	0.589
Pathologic T stage (≥pT3 vs < pT3)	2.994	1.762-5.088	**<0.001***	4.629	2.397-8.941	**<0.001***	3.320	2.005-5.497	**<0.001***
N stage (N1 vs N0)	2.054	1.810-5.205	**0.029***	1.849	1.190-4.953	**0.022***	1.289	1.007-3.095	**0.047***
Tumor grade (≥3 vs <3)	1.778	0.903-3.501	0.096	1.758	0.768-4.026	0.182	1.582	0.833-3.003	0.161
LVI (Yes vs No)	1.120	0.512-2.449	0.777	1.145	0.515-2.543	0.740	1.224	0.618-2.423	0.562

Note: *statistically significant

Supplementary 4.

Table S4 Multivariate analysis of variables for the prediction of survival outcomes in training and validation cohorts when only including SII

Variables	Overall survival			Cancer-specific survival			Recurrence-free survival		
	HR	95%CI	*P* value	HR	95%CI	*P* value	HR	95%CI	*P* value
**Training cohort**									
Age (>65 vs ≤65 years)	2.178	1.342-3.535	**0.002***	1.883	1.098-3.231	**0.022***	1.604	1.037-2.483	**0.034***
SII (≥672.44 vs<672.44)	1.758	1.022-3.354	**0.047***	2.341	1.065-5.145	**0.034***	1.575	1.024-3.013	**0.037***
NLR (≥2.53 vs<2.53)	0.867	0.425-1.766	0.694	0.559	0.240-1.304	0.179	0.590	0.301-1.154	0.123
PLR (≥126.88 vs<126.88)	1.718	0.971-3.039	0.063	1.688	0.893-3.192	0.107	1.468	0.872-2.471	0.148
MLR (≥0.35 vs<0.35)	0.898	0.548-1.473	0.670	0.919	0.522-1.616	0.769	1.031	0.635-1.676	0.901
Tumor size (≥3 vs<3)	1.666	1.083-2.563	**0.020***	1.739	1.068-2.831	**0.026***	1.451	0.967-2.177	0.072
Pathologic T stage (≥pT3 vs < pT3)	2.515	1.536-4.119	**<0.001***	2.825	1.590-5.017	**<0.001***	1.699	1.046-2.759	**0.032***
N stage (N1 vs N0)	2.233	1.008-4.947	**0.048***	1.964	1.168-4.445	**0.035***	2.217	1.006-4.884	**0.048***
Tumor grade (≥3 vs <3)	1.386	0.647-2.972	0.401	2.047	0.717-5.846	0.181	1.522	0.803-2.882	0.198
LVI (Yes vs No)	1.642	0.798-3.377	0.178	2.218	1.041-4.726	**0.039***	1.518	0.742-3.105	0.253
**Validation cohort**									
Age (>65 vs ≤65 years)	1.220	0.762-1.953	0.407	0.992	0.592-1.662	0.975	1.075	0.690-1.674	0.749
SII (≥672.44 vs<672.44)	1.894	1.110-3.942	**0.038***	2.599	1.121-6.025	**0.026***	1.483	1.016-2.892	**0.047***
NLR (≥2.53 vs<2.53)	0.816	0.381-1.748	0.602	0.779	0.321-1.889	0.581	0.994	0.500-1.973	0.985
PLR (≥126.88 vs<126.88)	1.572	0.880-2.810	0.127	1.426	0.763-2.662	0.266	1.374	0.777-2.429	0.275
MLR (≥0.35 vs<0.35)	1.651	0.975-2.798	0.062	1.534	0.859-2.737	0.148	1.413	0.856-2.332	0.177

Tumor size (≥3 vs<3)	1.240	0.790-1.945	0.349	1.282	0.773-2.125	0.336	1.198	0.774-1.852	0.418
Pathologic T stage (≥pT3 vs < pT3)	3.133	1.858-5.282	**<0.001***	4.741	2.479-9.068	**<0.001***	3.361	2.039-5.538	**<0.001***
N stage (N1 vs N0)	2.150	1.053-5.417	**0.035***	1.831	1.016-4.885	**0.047***	1.303	1.045-3.120	**0.032***
Tumor grade (≥3 vs <3)	1.903	0.978-3.700	0.058	1.884	0.835-4.251	0.127	1.595	0.845-3.010	0.149
LVI (Yes vs No)	0.953	0.440-2.066	0.903	0.993	0.453-2.181	0.987	1.156	0.588-2.274	0.674

Note: *statistically significant

Supplementary 5.

Table S5 Multivariate analysis of variables for the prediction of survival outcomes in training and validation cohorts when only including PNI

Variables	Overall survival			Cancer-specific survival			Recurrence-free survival		
	HR	95%CI	*P* value	HR	95%CI	*P* value	HR	95%CI	*P* value
**Training cohort**									
Age (>65 vs ≤65 years)	2.033	1.252-3.301	**0.004***	1.728	1.009-2.958	**0.046***	1.508	0.974-2.335	0.066
NLR (≥2.53 vs<2.53)	1.171	0.669-2.049	0.581	0.926	0.496-1.728	0.808	0.735	0.439-1.229	0.240
PLR (≥126.88 vs<126.88)	1.687	0.971-2.932	0.063	1.718	0.921-3.203	0.089	1.443	0.877-2.376	0.149
MLR (≥0.35 vs<0.35)	0.788	0.479-1.297	0.349	0.778	0.442-1.368	0.384	0.949	0.584-1.543	0.833
PNI (<47.83 vs≥47.83)	1.799	1.126-2.873	**0.014***	1.858	1.085-3.182	**0.024***	1.779	1.135-2.787	**0.012***
Tumor size (≥3 vs<3)	1.686	1.098-2.588	**0.017***	1.709	1.056-2.768	**0.029***	1.484	0.989-2.226	0.056
Pathologic T stage (≥pT3 vs < pT3)	2.547	1.548-4.190	**<0.001***	2.884	1.615-5.150	**<0.001***	1.678	1.027-2.741	**0.039***
N stage (N1 vs N0)	2.304	1.033-5.140	**0.041***	2.026	1.087-4.627	**0.044***	2.382	1.075-5.280	**0.033***
Tumor grade (≥3 vs <3)	1.421	0.665-3.034	0.365	2.092	0.736-5.947	0.166	1.563	0.827-2.955	0.169
LVI (Yes vs No)	1.644	0.803-3.368	0.174	2.245	1.057-4.769	**0.035***	1.389	0.675-2.858	0.372
**Validation cohort**									
Age (>65 vs ≤65 years)	1.041	0.640-1.694	0.871	0.877	0.514-1.499	0.632	1.005	0.637-1.586	0.983
NLR (≥2.53 vs<2.53)	1.004	0.556-1.815	0.988	1.272	0.648-2.498	0.484	1.169	0.673-2.031	0.580
PLR (≥126.88 vs<126.88)	1.974	1.017-3.319	0.065	1.988	0.889-3.516	0.118	1.599	0.962-2.657	0.070
MLR (≥0.35 vs<0.35)	1.424	0.835-2.428	0.194	1.287	0.717-2.311	0.397	1.320	0.794-2.193	0.284
PNI (<47.83 vs≥47.83)	2.134	1.181-3.856	**0.012***	1.783	1.118-3.462	**0.038***	1.361	1.004-2.275	**0.042***
Tumor size (≥3 vs<3)	1.083	0.680-1.725	0.737	1.103	0.651-1.869	0.716	1.115	0.713-1.744	0.634
Pathologic T stage (≥pT3 vs < pT3)	3.258	1.935-5.487	**<0.001***	5.197	2.713-9.955	**<0.001***	3.471	2.112-5.703	**<0.001***
N stage (N1 vs N0)	1.945	1.166-4.937	**0.042***	1.689	1.027-4.547	**0.043***	1.259	1.023-3.032	**0.047***
Tumor grade (≥3 vs <3)	1.615	0.825-3.159	0.162	1.548	0.681-3.518	0.297	1.465	0.777-2.764	0.238
LVI (Yes vs No)	1.061	0.487-2.311	0.882	1.073	0.485-2.372	0.863	1.195	0.605-2.362	0.608

Note: *statistically significant

Supplementary 6.

Figure S1. Kaplan-Meier curves for OS, CSS, and RFS in UTUC patients stratified by SII, NLR,

PLR, MLR, and PNI in the validation cohort.

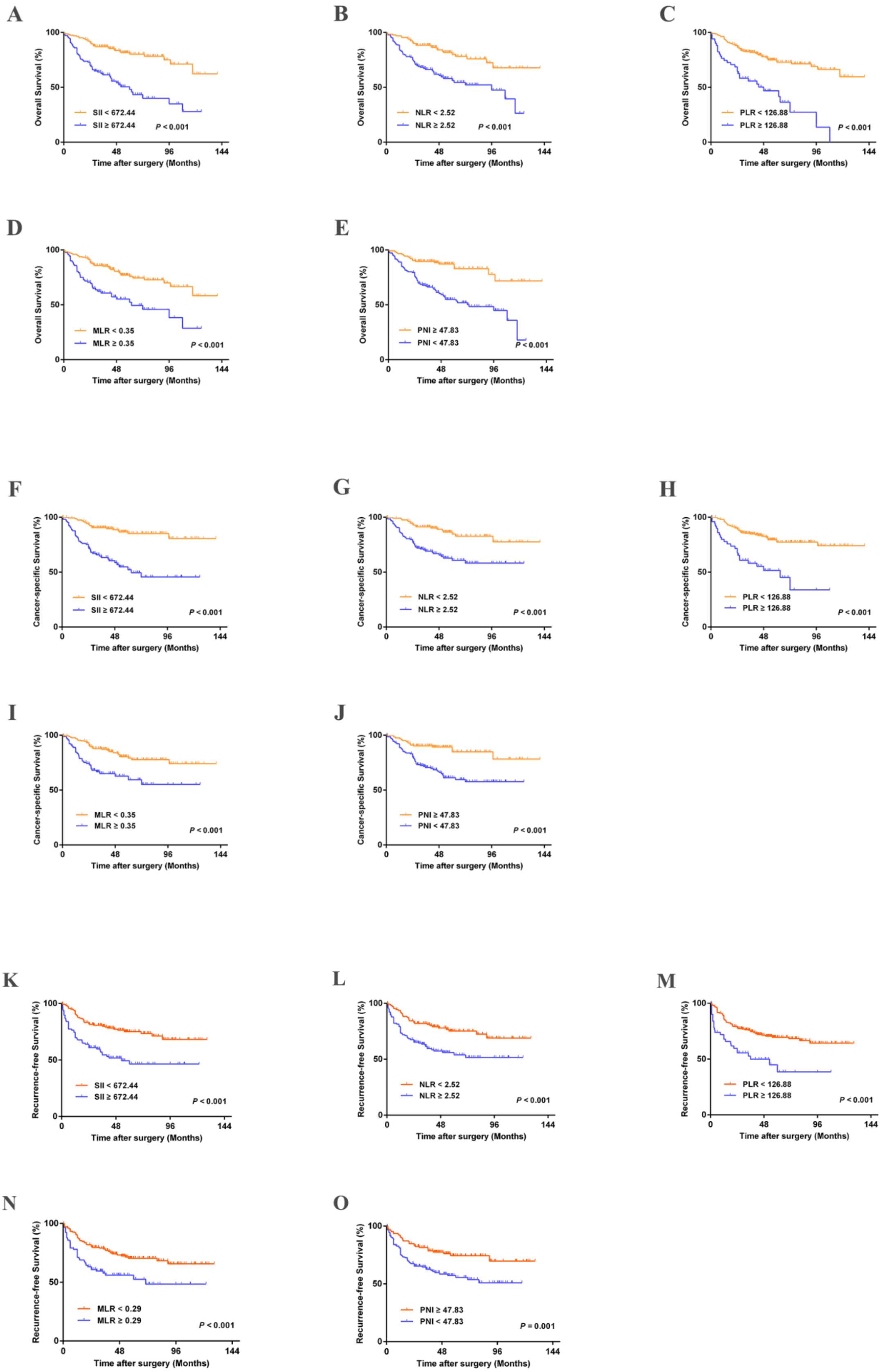
A
Overall Survival (%)
SII < 672.44
SII ≥ 672.44
P < 0.001
Time after surgery (Months)
B
Overall Survival (%)
NLR < 2.52
NLR ≥ 2.52
P < 0.001
Time after surgery (Months)
C
Overall Survival (%)
PLR < 126.88
PLR ≥ 126.88
P < 0.001
Time after surgery (Months)
D
Overall Survival (%)
MLR < 0.35
MLR ≥ 0.35
P < 0.001
Time after surgery (Months)
E
Overall Survival (%)
PNI ≥ 47.83
PNI < 47.83
P < 0.001
Time after surgery (Months)
F
Cancer-specific Survival (%)
SII < 672.44
SII ≥ 672.44
P < 0.001
Time after surgery (Months)
G
Cancer-specific Survival (%)
NLR < 2.52
NLR ≥ 2.52
P < 0.001
Time after surgery (Months)
H
Cancer-specific Survival (%)
PLR < 126.88
PLR ≥ 126.88
P < 0.001
Time after surgery (Months)
I
Cancer-specific Survival (%)
MLR < 0.35
MLR ≥ 0.35
P < 0.001
Time after surgery (Months)
J
Cancer-specific Survival (%)
PNI ≥ 47.83
PNI < 47.83
P < 0.001
Time after surgery (Months)
K
Recurrence-free Survival (%)
SII < 672.44
SII ≥ 672.44
P < 0.001
Time after surgery (Months)
L
Recurrence-free Survival (%)
NLR < 2.52
NLR ≥ 2.52
P < 0.001
Time after surgery (Months)
M
Recurrence-free Survival (%)
PLR < 126.88
PLR ≥ 126.88
P < 0.001
Time after surgery (Months)
N
Recurrence-free Survival (%)
MLR < 0.29
MLR ≥ 0.29
P < 0.001
Time after surgery (Months)
O
Recurrence-free Survival (%)
PNI ≥ 47.83
PNI < 47.83
P = 0.001
Time after surgery (Months)

Supplementary 7.

Figure S2. Kaplan-Meier analysis for urologic outcomes in UTUC patients based on SII-PNI in the validation cohort.

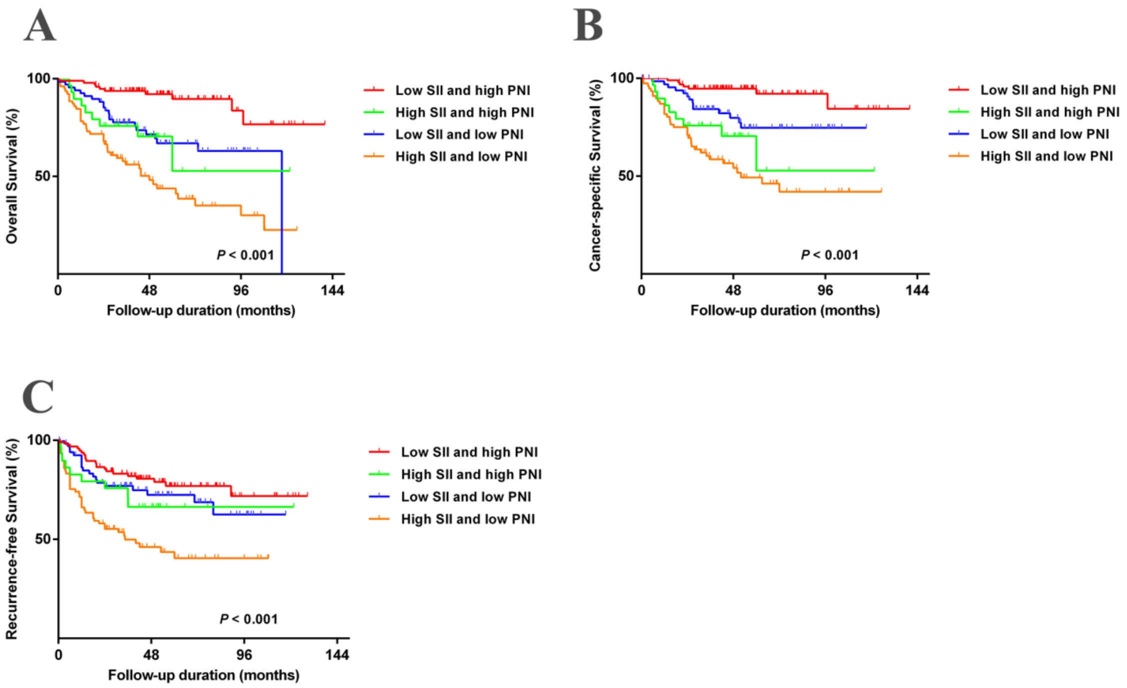

Supplementary 8

Figure S3. Postoperative nomograms to predict the probability of OS (A) rates in patients with UTUC after surgery and Calibration curve for predicting 3- and 5-year survival of OS (B and C) in the validation cohort.

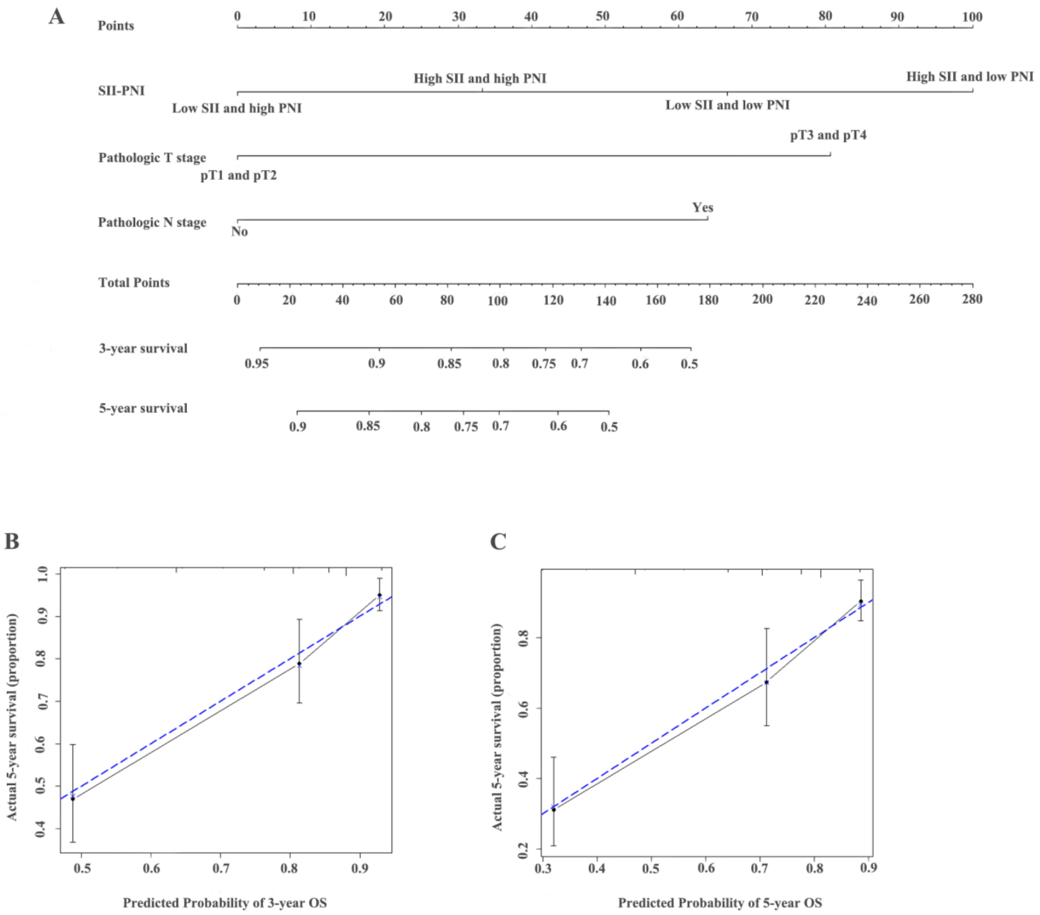

Supplementary 9

Figure S4. Postoperative nomograms to predict the probability of CSS (A) rates in patients with UTUC after surgery and Calibration curve for predicting 3- and 5-year survival of CSS (B and C) in the validation cohort.

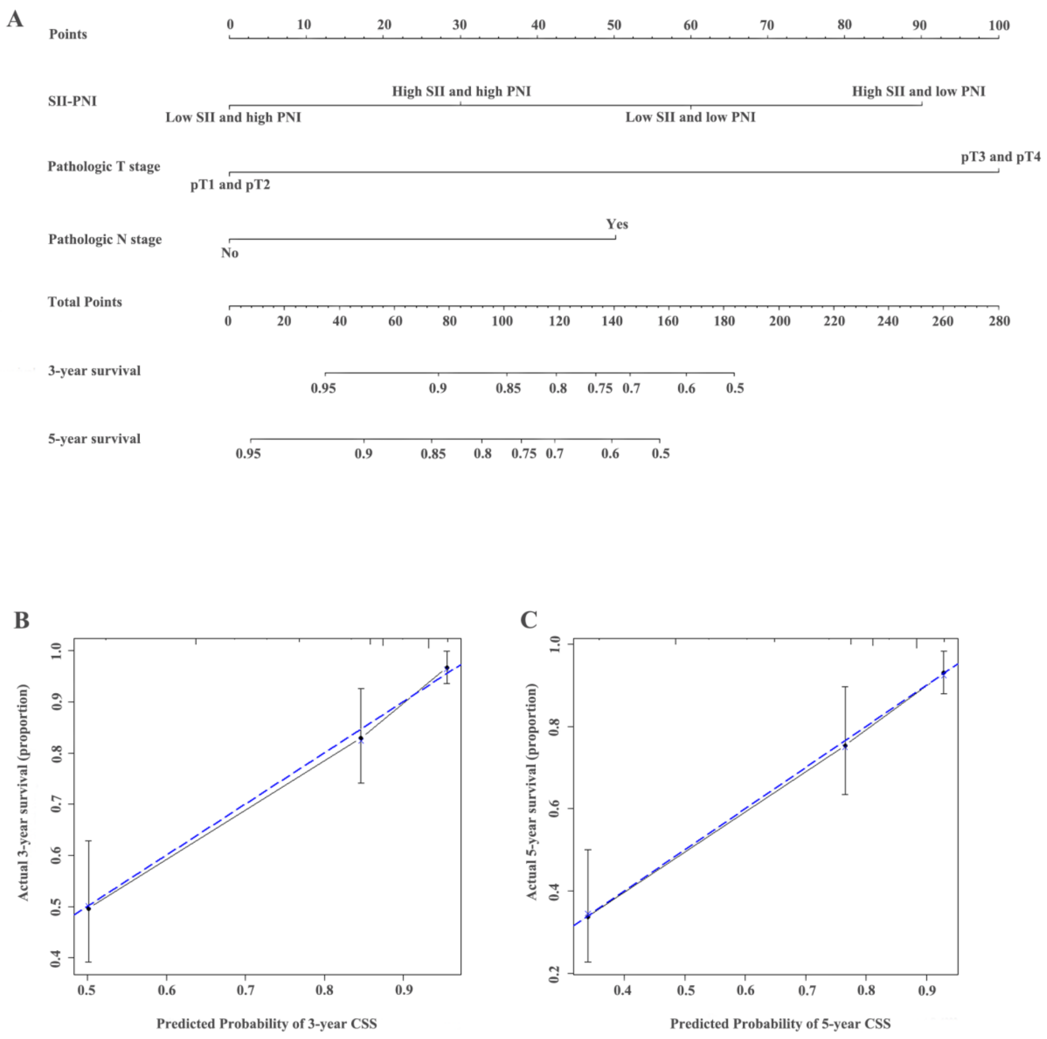

Supplementary 10

Figure S5. Postoperative nomograms to predict the probability of RFS (A) rates in patients with UTUC after surgery and Calibration curve for predicting 3- and 5-year survival of RFS (B and C) in the validation cohort.

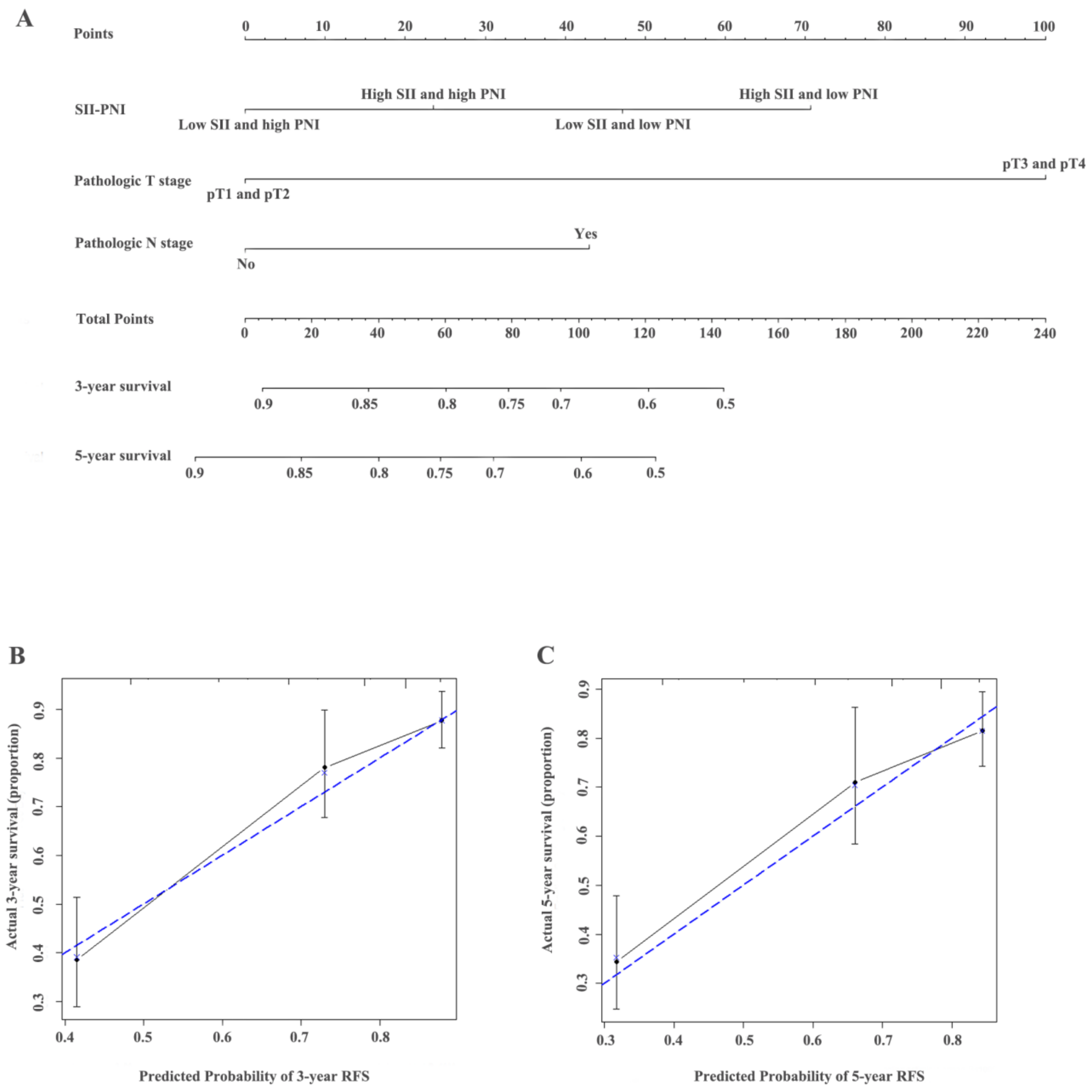
A
Points
0 10 20 30 40 50 60 70 80 90 100
SII-PNI
High SII and high PNI
High SII and low PNI
Low SII and high PNI
Low SII and low PNI
Pathologic T stage
pT3 and pT4
pT1 and pT2
Pathologic N stage
Yes
No
Total Points
0 20 40 60 80 100 120 140 160 180 200 220 240
3-year survival
0.9 0.85 0.8 0.75 0.7 0.6 0.5
5-year survival
0.9 0.85 0.8 0.75 0.7 0.6 0.5
B
Actual 3-year survival (proportion)
0.3 0.4 0.5 0.6 0.7 0.8 0.9
0.4 0.5 0.6 0.7 0.8
Predicted Probability of 3-year RFS
C
Actual 5-year survival (proportion)
0.3 0.4 0.5 0.6 0.7 0.8 0.9
0.3 0.4 0.5 0.6 0.7 0.8
Predicted Probability of 5-year RFS
